# The impact of COVID-19 pandemic on the rate of newly diagnosed gynecological and breast cancers: a tertiary center perspective

**DOI:** 10.1007/s00404-021-06259-5

**Published:** 2021-09-24

**Authors:** Katharina Knoll, Elisabeth Reiser, Katharina Leitner, Johanna Kögl, Christoph Ebner, Christian Marth, Irina Tsibulak

**Affiliations:** 1 Department of Obstetrics and Gynecology, Medical University Innsbruck, Anichstrasse 35, Innsbruck, Austria; 2 Department of Gynecological Endocrinology and Reproductive Medicine, Medical University Innsbruck, Innsbruck, Austria

**Keywords:** Gynecology, Carcinoma, Neoplasms, Breast cancer, COVID-19

## Abstract

**Purpose:**

The aim of the present study was to assess the impact of postponed screening examinations and lockdown measures on gynecological and breast cancer diagnoses throughout the year 2020 in a gynecological oncological center in Austria.

**Methods:**

Data of 889 patients with either newly diagnosed gynecological or breast cancer between January 2019 and December 2020 were collected. Clinical parameters including symptoms, performance status, comorbidities and referral status were compared in patients, who were newly diagnosed with cancer in the period of the first lockdown from March 2020 to April 2020 and the second lockdown from November 2020 to December 2020 and compared to the same period in 2019.

**Results:**

Our results showed a strong decline in newly diagnosed cancers during the lockdown periods: −45% in gynecological cancer and -52% in breast cancer compared to the same period in 2019. Compared to the analogue period of 2019, breast cancer patients reported significantly more tumor-associated symptoms (55% vs. 31%, *p* = 0.013) during and in between (48% vs. 32%, *p* = 0.022) the lockdowns. During the lockdown, periods in the group of breast cancer patients’ tumor stage varied significantly compared to 2019 (T2–T4; *p* = 0.047).

**Conclusion:**

Both lockdowns led to a strong decrease in newly diagnosed gynecological and breast cancers. Treatment delays in potentially curable disease could lead to inferior clinical outcomes, with the risk of missing the optimal treatment window. As the COVID-19 pandemic will be a challenge for some time to come, new strategies in patient care are needed to optimize cancer screening and management during the pandemic.

## Introduction

The ongoing coronavirus disease 2019 (COVID-19) pandemic remains a challenge for most countries around the world, affecting not only their healthcare system, but also economic development, socio-political attitude, educational and cultural sectors. As of 26 January 2021, more than 100 million infections were reported worldwide with a death toll of more than 2 million [1]. In Austria, more than 402 000 cases and 7416 deaths were confirmed by 26 January 2020, leading to a death rate of 1.8% [2].

The first two patients in Innsbruck, Austria, were diagnosed with (COVID-19) on 25 February 2020. Within few weeks, the number of cases increased throughout the country, leading the government to the implementation of a nationwide hard lockdown on 16 March 2020, closing all non-essential businesses and switching to distance learning in schools and universities. During the first wave of the COVID-19 pandemic, Austria’s governmental measures prevented an overload of the healthcare system, as the maximum intensive care bed occupancy only reached 26% [2]. By the middle of April, the stay-at-home orders started to be loosened. The first lockdown ended on April 30^st^ enabling most of public life by the beginning of May. However, regained freedom of travel and growing get-together trend led to a new escalation of newly diagnosed COVID-19 cases, so that a nationwide curfew was announced on 21 September, followed by the second lockdown enforced from 3 November.

In Austria, general practitioners and specialists play a central role in preventive and primary healthcare and they refer patients to hospitals, if necessary. Gynecological screening examinations and routine checkups in Austria include annual PAP smear testing and biennial mammography. During the first lockdown, these screening examinations and routine checkups were disrupted to prevent the spread of the virus and to increase the hospitals’ bed capacity across the country [3]. These restrictions directly affected the tertiary centre as less patients were referred to the department with suspicious results and this led to a strong decline in newly diagnosed gynaecological and breast cancers in Austria, as already reported (−24% in March 2020 vs. March 2019,  − 49% in April 2020 vs. April 2019,  − 49% in May 2020 vs. May 2019) [3]. Such a dramatic decrease in cancer diagnoses reflects the failures of pandemic management and, therefore, underlines the pivotal role of the gynecological screening examinations and routine checkups. After the first lockdown, significant efforts were made to relaunch health prevention and to promote the importance of screening programs during the pandemic. Furthermore, the safety of patients in hospitals was improved, as mandatory COVID-19 testing was implemented for every admitted patient as well as weekly screening of healthcare workers. During the second lockdown, screening examinations and routine checkups (PAP, biennial mammography) were maintained and access to the hospital, general practitioners and specialists was controlled by COVID-19 rapid testing and temperature measurements.

The aim of this study was to assess the impact of the COVID-19 pandemic and its management throughout the year 2020 on the rate of newly diagnosed gynecological and breast cancers from the perspective of a tertiary referral center in Innsbruck (federal state of Tyrol, Austria).

## Materials and methods

### Patients

All patients diagnosed with gynecological or breast cancer at the Department of Obstetrics and Gynecology, Medical University of Innsbruck in the period from January 2019 to December 2020, were included in the present study. The primary endpoint was the rate of newly diagnosed gynecological and breast cancer during the lockdown periods in 2020 and during the same time period in 2019. Gynecological cancer was defined as one of the following diagnoses: ovarian or tubal carcinoma, cervical carcinoma, endometrial carcinoma, uterine sarcoma, vulvar carcinoma, vaginal carcinoma, and germ cell tumors. Ductal carcinoma in situ (DCIS) of the breast was included in the breast cancer cohort. Clinical parameters such as tumor-specific symptoms, performance status, leading comorbidities, tumor type, tumor stage, referral status and COVID-19 infection status were the secondary endpoints. Referral status was defined as followed: referral by a specialist with or without symptoms, or self-determined presentation at emergency or gynecological department with or without tumor-specific symptoms. Leading comorbidities were classified as: cardiovascular, malignant, respiratory, infectious, rheumatic, endocrine and metabolic, psychiatric disease and others. Frontline therapy included surgery, neoadjuvant chemotherapy, neoadjuvant endocrine therapy, radiotherapy, chemoradiation and palliative therapy. Tumor stage was defined according to the Fédération Internationale de Gynécologie et d'Obstétrique (FIGO) staging system for gynecological tumors and according to TNM staging system for breast cancer.

The following symptoms have been regarded as tumor-associated: abdominal swelling/pain, abnormal vaginal bleeding (postmenopausal bleeding, menorrhagia, metrorrhagia, spotting) and palpable mass of the breast.

To assess the impact of the COVID-19 lockdown measures on the rate of newly diagnosed gynecological and breast tumors.

Subgroup analyses were performed in patients, who were newly diagnosed with cancer during the lockdown periods (the first lockdown from 16 March 2020 to 30 April 2020 and the second lockdown from 3 November 2020 to 31 December 2020) and compared to the same period in 2019 (16 March 2019 to 30 April 2019 and 3 November 2019 to 31 December 2019), respectively. Gynecological and breast cancers were evaluated separately for better clarity, as breast cancer cases are the vast majority of cancer diagnoses at our department.

### Statistical analyses

Descriptive statistics, such as median, mean, frequencies and percentages, were used to describe the data. Clinical characteristics were analyzed using χ^2^ and Mann–Whitney *U* tests. For all statistical analyses, SPSS software version 26.0 was used. Two-sided *p* values below 0.05 were regarded as significant.

### Ethical approval

The ethics committee of the Medical University of Innsbruck, Austria approved the present study (IRB-Number 1163/2020).

## Results

In this study, 889 patients (495 and 394 in 2019 and 2020, respectively) with either newly diagnosed breast (*n* = 596) or gynecological (*n* = 293) cancer were included.

During the lockdowns, screening examinations and hospital bed capacity were reduced. This led to a strong decline of newly diagnosed cancers during the first and second lockdown: −45% in gynecological cancer and -52% in newly diagnosed breast cancer compared to the same period in 2019.

Patients’ characteristics, tumor type and stage as well as clinical parameters are presented for breast cancer in Table 1 and for gynecological tumors in Table 2.

**Table 1:** Breast cancer patients’ characteristics

Parameter	16 Mar –30 Apr and 3 Nov–31 Dec 2019	16 Mar –30 Apr and 3 Nov–31 Dec 2020	Difference (%)	*p* value	1 May–2 Nov 2019	1 May–2 Nov 2020	Difference (%)	*p* value
Breast cancer patients diagnosed								
Number of patients	115	55	− 52%		148	157	+6%	
Age, median (range)	63 (32–90)	60 (28–93)	− 5%	*p *= 0.145*	59 (21–90)	62 (25–96)	+ 5%	*p* = 0.035*
Reported tumor-associated symptoms at diagnosis								
No	78 (68%)	25 (45%)	− 23%	***p*** *= ***0.013**^†^	*98 (67%)*	*81 (51%)*	*− 16%*	***p = *0.022**^†^
Yes	36 (31%)	30 (55%)	+24%		48 (32%)	75 (48%)	+16%	
Unknown	1 (1%)	0 (0%)			2 (1%)	1 (1%)		
Referral of patients								
Patients themselves without symptoms	4 (3%)	1 (2%)	− 1%	*p* = 0.288^†^	*7 (5%)*	*8 (5%)*	*0%*	*p* = 0.371^†^
Patients themselves with symptoms	13 (11%)	12 (21%)	+10%		11 (8%)	16 (10%)	+2%	
Specialist	94 (82%)	40 (73%)	-9%		123 (83%)	131 (84%)	+1%	
General practitioner	3 (3%)	1 (2%)	− 1%		5 (3%)	2 (1%)	− 2%	
Emergency	0 (0%)	1 (2%)	+2%		0 (0%)	0 (0%)	0%	
Unknown	1 (1%)	0 (0%)			2 (1%)	0 (0%)		
Leading comorbidities								
None	55 (47%)	35 (64%)	+17%	*p* = 0.077^†^	84 (57%)	83 (53%)	− 4%	*p* = 0.117^†^
Cardiovascular disease	28 (24%)	10 (18%)	-6%		22 (15%)	38 (24%)	+9%	
Malignant disease	6 (5%)	5 (9%)	+4%		13 (9%)	14 (9%)	0%	
Respiratory disease	4 (4%)	0 (0%)	− 4%		6 (4%)	8 (5%)	+1%	
Infectious disease	0 (0%)	0 (0%)	0%		4 (3%)	2 (1%)	− 2%	
Rheumatic disease	2 (2%)	1(2%)	0%		0 (0%)	0 (0%)	0%	
Endocrine and metabolic	16 (14%)	1 (2%)	-12%		15 (10%)	6 (4%)	− 6%	
Psychiatric disorders	2 (2%)	3 (5%)	+3%		3 (2%)	3 (2%)	0%	
Others	1 (1%)	0 (0%)	− 1%		1 (1%)	0 (0%)	− 1%	
Unknown	1 (1%)	0 (0%)			0 (0%)	3 (2%)		
Frontline therapy								
Surgery	82 (71%)	33 (60%)	− 11%	*p* = 0.456^†^	97 (66%)	98 (62%)	− 4%	*p *= 0.736^†^
Neoadjuvant chemotherapy	20 (17%)	15 (27%)	+10%		30 (20%)	35 (22%)	+2%	
Neoadjuvant endocrine therapy	10 (9%)	5 (9%)	0%		15 (10%)	19 (12%)	+2%	
Radiotherapy	0 (0%)	0 (0%)	0%		0 (0%)	0 (0%)	0%	
Palliative therapy	3 (3%)	2 (4%)	+1%		6 (4%)	4 (3%)	− 1%	
Tumor stage								
Tis	10 (9%)	2 (4%)	-5%	***p* = 0.047**^†^	14 (9%)	11 (7%)	− 2%	*p* = 0.708^†^
T1	72 (62%)	24 (43%)	− 19%		78 (53%)	81 (52%)	− 1%	
T2	17 (15%)	13 (24%)	+9%		34 (23%)	33 (21%)	− 2%	
T3	7 (6%)	6 (11%)	+5%		13 (9%)	23 (15%)	+6%	
T4	1 (1%)	3 (6%)	+5%		7 (5%)	7 (4%)	− 1%	
Unknown	8 (7%)	7 (12%)			2 (1%)	2 (1%)		

16 March 2020–30 April 2020 and 3 November 2020–31 December 2020 refers to the lockdown periods 1 and 2, respectively. 1 May 2020–2 November 2020 refers to the time between the two lockdowns

*Mann–Whitney *U* test

^†^χ2 test

**Table 2:** Gynecological cancer patients’ characteristics

Parameter	16 Mar–30 Apr 30 and 3 Nov–31 Dec 2019	16 Mar–30 Apr 30 and 3 Nov–31 Dec 2020	Difference (%)	*p* value	1 May–2 Nov 2019	1 May–2 Nov 2020	Difference (%)	*p* value
Gynecological cancer patients diagnosed								
Number of patients	29	16	− 45%		102	57	− 44%	
Age, median (range)	60 (34–83)	66 (28–93)	+ 10%	*p* = 0.164*	61 (25–96)	62 (30–95)	+ 1%	*p* = 0.568*
Reported tumor-associated symptoms at diagnosis								
No	5 (17%)	1 (6%)	− 11%	*p* = 0.426^†^	*23 (22%)*	*19 (33%)*	+ 11%	*p* = 0.170^†^
Yes	16 (55%)	12 (75%)	+ 20%		60 (59%)	34 (60%)	+ 1%	
Unknown	8 (28%)	3 (19%)			19 (19%)	4 (7%)		
Referral of patients								
Patients themselves without symptoms	1 (3%)	0 (0%)	− 3%	*p* = 0.118^†^	0 (0%)	0 (0%)	0%	***p* < 0.001**^†^
Patients themselves with symptoms	0 (0%)	0 (0%)	0%		3 (3%)	16 (28%)	+ 25%	
Specialist	20 (69%)	13 (81%)	+ 12%		79 (78%)	40 (70%)	− 8%	
General practitioner	2 (8%)	3 (19%)	+ 11%		6 (6%)	1 (2%)	− 4%	
Emergency	3 (10%)	0 (0%)	− 10%		2 (2%)	0 (0%)	− 2%	
Unknown	3 (10%)	0 (0%)			12 (11%)	0 (0%)		
Comorbidities								
None	6 (20%)	3 (19%)	− 1%	*p* = 0.751^†^	28 (28%)	26 (46%)	-18%	***p*** = **0.010**^†^
Yes	16 (55%)	11 (69%)	− 14%		53 (52%)	29 (51%)	-1%	
Unknown	7 (25%)	2 (12%)			21 (20%)	2 (3%)		
Frontline therapy								
Operation	22 (75%)	10 (63%)	− 12%	*p* = 0.559^†^	68 (66%)	43 (74%)	+ 8%	*p* = 0.677^†^
Neoadjuvant chemotherapy	3 (11%)	3 (19%)	+ 8%		17(17%)	8 (14%)	− 3%	
Neoadjuvant endocrine therapy	0 (0%)	0 (0%)	0%		0 (0%)	0 (0%)	0%	
Radiation	0 (0%)	0 (0%)	0%		2 (2%)	1 (2%)	0%	
Combined chemoradiation	3 (11%)	1 (6%)	-5%		11 (11%)	2 (4%)	− 7%	
COVID associated treatment delay	0 (0%)	0 (0%)	0%		0 (0%)	0 (0%)	0%	
Palliative therapy	0 (0%)	1 (6%)	+ 6%		3 (3%)	2 (4%)	+ 1%	
Unknown	1 (3%)	1 (6%)			1 (1%)	1 (2%)		
Diagnosis								
Ovarian cancer	13 (45%)	9 (56%)	+ 11%	*p* = 0.542^†^	40 (39%)	23 (40%)	+ 1%	*p* = 0.558^†^
Endometrial cancer	7 (24%)	6 (38%)	+ 14%		29 (28%)	19 (33%)	+ 5%	
Cervical cancer	5 (18%)	1 (6%)	− 12%		19 (19%)	5 (9%)	− 10%	
Vulva cancer	2 (7%)	0 (0%)	− 7%		6 (6%)	6 (10%)	+ 4%	
Sarcoma	1 (3%)	0 (0%)	− 3%		4 (4%)	2 (4%)	0%	
Others	1 (3%)	0 (0%)	− 3%		4 (4%)	2 (4%)	0%	
FIGO stage ovarian cancer								
I	3 (23%)	1 (11%)	− 12%	*p* = 0.145^†^	12 (30%)	6 (26%)	− 4%	*p* = 0.795^†^
II	1 (7%)	0 (0%)	− 7%		1 (3%)	1 (4%)	+ 1%	
III	7 (55%)	5 (56%)	+ 1%		19 (48%)	10 (44%)	− 4%	
IV	2 (15%)	0 (0%)	− 15%		6 (15%)	6 (26%)	+ 11%	
Unknown	0 (0%)	3 (33%)			2 (4%)	0 (0%)		
FIGO stage endometrial cancer								
I	7 (100%)	5 (83%)	− 17%	*p* = 0.261^†^	23 (80%)	16 (85%)	+ 5%	*p* = 0.218^†^
II	0 (0%)	0 (0%)	0%		1 (3%)	2 (10%)	+ 7%	
III	0 (0%)	0 (0%)	0%		0 (0%)	1 (5%)	+ 5%	
IV	0 (0%)	0 (0%)	0%		3 (10%)	0 (0%)	-10%	
Unknown	0 (0%)	1 (17%)			2 (7%)	0 (0%)		

16 March 2020–30 April 2020 and 3 November 2020–31 December 2020 refers to the lockdown periods 1 and 2, respectively. 1 May 2020–2 November 2020 refers to the time between the two lockdowns

*Mann–Whitney *U *test

^†^χ^2^ test

**Fig. 1:**
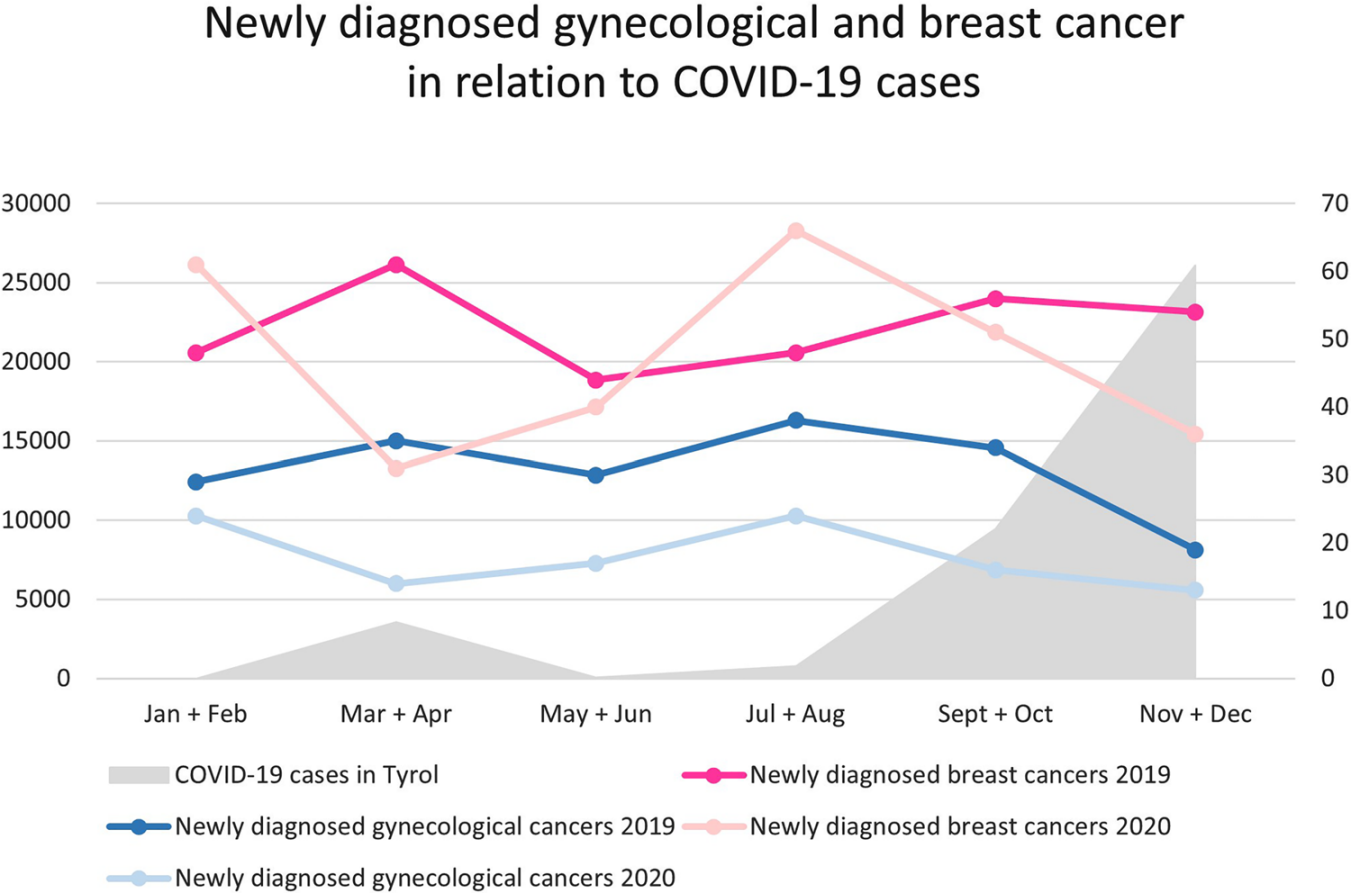
Impact of COVID-19 lockdown on newly diagnosed cancers. Numbers of newly diagnosed breast and gynecological cancers per month in 2020 compared to the same periods in 2019. Pre-lockdown refers to 1 January–15 March, lockdown 1 to 16 March– 30 April, between lockdowns to 1 May–2 November and lockdown 2 to 3 November–31 December

Analysis of 2019 and 2020 revealed a decrease of 70 and 35%, respectively, of newly diagnosed gynecological and breast cancers in particular during the first lockdown (Fig. 1). According to the above, the number of newly diagnosed gynecological cancer cases was remarkably lower in 2020 compared to 2019 during the first lockdown period (−45%) as well as between the two lockdowns (−44%). An inverse correlation was found between the number of positively tested COVID-19 patients in the federal state of Tyrol (Austria) and newly diagnosed breast cancer cases at our department during 2020.

### Sub-analysis of breast cancer patients

Compared to the analogue period of 2019, breast cancer patients reported significantly more tumor-associated symptoms (55% vs. 31%, *p* = 0.013) during and in between (48% vs. 32%, *p* = 0.022) the lockdowns (Table 1). In contrast to 2019, patients were less likely to be referred by a specialist, whereas an increase of self-referring patients, due to symptoms, was noted. Furthermore, patients’ tumor stage during the lockdown varied significantly compared to 2019 (*p* = 0.047). Despite being insignificant, fewer patients underwent immediate surgery (−11%), whereas an increase of neoadjuvant chemotherapy (+ 10%) was indicated in this sub-cohort (Table 1). The drop of newly diagnosed cancer cases during first lockdown was followed by an increased number of diagnosis in between the lockdowns (+ 6%).

### Sub-analysis of gynecological cancer patients

As seen in the breast cancer subgroup, an increase of patients presenting themselves with tumor-associated symptoms (+ 20%, *p* = 0.384) was noted during the lockdown, despite being insignificant (Table 2). Between the two lockdown periods, a significant leap (+ 25%) of self-referring symptomatic patients was observed (*p* < 0.001) (Table 2).

Overall, the tumor stage did not differ significantly between the two analyzed years (Table 2). However, a drop in newly diagnosed cervical cancer (18% vs. 6%) was observed. During the lockdown periods, a trend towards neoadjuvant chemotherapy (+ 8%; *p* = 0.677), as well as a non-significant decrease in primary surgical treatment (−12%; *p* = 0.559) was reported (Table 2).

A significant increase of patients without leading comorbidities was seen in the gynecological cancer cohort during the lockdowns (*p* = 0.010), whereas this change remained insignificant in the breast cancer subgroup (*p* = 0.102).

### COVID-19 screening

Routine preoperative COVID-19 screening was performed in 296 patients leading to the detection of 3 positive COVID-19 asymptomatic patients (1%). Of those, one was diagnosed with breast cancer and two with gynecological cancer (endometrial and ovarian cancer). For two of them, surgery had to be postponed for 3 weeks until they reached a negative test result. The third patient (ovarian cancer) was admitted to our department and undergone paracentesis but denied further treatment irrespective of her COVID-19 infection.

## Discussion

In the current study, we found a strong decline of newly diagnosed gynecological and breast cancer cases in 2020 as compared to 2019. This decrease was mainly due to a lower cancer detection rate at our department during the two lockdown periods (16 March 2020–30 April 2020 and 3 November 2020–31 December 2020) as compared to the same period in 2019.

To guarantee adequate patient care, considerable efforts have been made during the first lockdown to facilitate gynecological screening examinations with a minimized risk of a hospital acquired infection: COVID-19 symptom screening by questionnaires, temperature measurements, mandatory facemasks and optimized patients waiting time. By 14th of June, these actions were expanded by COVID-19 screening of every admitted patient. Therefore, although rising infection rates were experienced during fall and winter, screening examinations and outpatient care were guaranteed. However, as our study shows, even with optimized pandemic management, a strong decline in newly diagnosed cancers was still observed throughout the year 2020 as compared to 2019.

In the group of breast cancer, a strong decline of cancer detection rate was observed during both lockdown periods. In between the lockdowns, cases of newly diagnosed breast cancer increased and even reached a higher number as compared to 2019. We suppose that patients who missed screening examinations during the first lockdown, tended to catch up their appointment as soon as the pandemic situation looked stabilized. Significantly more patients with tumor-specific symptoms presented directly at our department or were referred by their gynecologist due to these symptoms. This observation indicates that tumor-specific symptoms forced patients to consult a doctor, while non-symptomatic cancer cases in patients who missed the routine mammography remained undetected. The observed tumor stage shift towards higher stage at diagnosis might also be explained by postponed mammography during the first lockdown. However, further studies on larger population are needed to prove this assumption. Moreover, there was an increasing trend towards neoadjuvant chemotherapy and a reduction in primary surgical cases during the lockdown periods. This might be explained by advanced tumor stage and the attempt to increase the overall hospitals’ bed capacity.

A decrease of 45% of newly diagnosed gynecological cancers was observed as compared to 2019. In particular, non-symptomatic tumors such as cervical cancer was underdetected in 2020, as annual PAP test was postponed during the first lockdown period. In the same time, tumor patients with tumor-specific symptoms such as postmenopausal bleeding or abdominal pain, less frequently consulted their specialist and presented themselves directly at our department.

Looking at percentages we have a similar loss in newly diagnosed tumors during the lockdown period in the breast cancer (−52%) as in the gynecological cancer group (−45%). In the breast cancer group, we have seen a strong decline in tumor diagnoses during the both lockdown periods and an increase in between the lockdowns. Whereas in gynecological cancer, we can just see a decline in percentage of cancer diagnoses throughout the year. The reason could be, that in gynecological cancer, we summarized different tumor entities and have a smaller sample size.

In accordance with our results, analyses of the national cancer screening patterns in the USA noticed a drop in breast and cervical cancer screening of 94% [4]. Among calculations, the COVID-19 pandemic could lead to 36 000 delayed or missed breast cancer diagnoses and 2 500 missed cases of cervical cancer in the three months from March to June 2020 [5]. Moreover, strong decline in newly diagnosed cancers was observed in breast, colorectal, lung and prostate cancer in the USA and across Europe [6–9]. Kaufman et al. have shown a decrease in mean weekly numbers of newly diagnosed patients with colorectal cancer, lung cancer, pancreatic cancer, gastric cancer, esophageal cancer and breast cancer of 46.4% for the timespan of 1 March to 18 April of 2020 when compared to the weekly numbers between 6 January 2019 and 29 February 2020. For breast cancer alone, they describe a decrease of 51.8% in the same period [10]. Also, Morais et al. were able to show a decrease of nearly 40% in new cases of 12 different types of cancer including breast cancer and cervical cancer. In their study, cervical cancer has shown the highest decrease with 74.3% when compared to the year 2019. For breast cancer, a decrease of 38.6% was found [11].

In contrast to most other published literature, this study includes data of the whole year 2020, compared to 2019. By investigating the whole year, we were able to find an inverse correlation of the number of positively tested COVID-19 patients in Austria and newly diagnosed cancers at our department. Thus, we analysed not only the differences in cancer diagnoses during the lockdown, but also investigated potential short term increases in between the lockdowns, as shown in our sub-analysis for breast cancer patients.

Efforts have been taken to organize cancer screening and management during the pandemic including the implementation of telemedicine for the outpatient treatment of cancer survivors to minimize face-to-face appointments [12]. It is recommended to continue oncological surgery, chemotherapy and radiotherapy based on priorities, while surgeries due to benign diseases should be postponed [13].

Treatment delays in potentially curable disease could lead to inferior outcomes and have impact on the overall survival of our patients, with the risk of missing the optimal treatment window. As the COVID-19 pandemic will be a challenge for some time to come, new strategies in patient care are needed to minimize the risk of infection. New strategies may include telemedicine or self-sampling HPV test, which detects viral nucleic acid rather than morphological changes and thus does not rely on healthcare practitioners visualizing the cervix [14], and thereby provide early diagnosis and improved treatment options for our patients. Most importantly, awareness must be raised for the importance of screening examinations to avoid any further shift in tumor stages at the time of diagnosis.

The major limitations of the current study are its relatively small sample size of only 889 patients and its single-center observational character. Despite these limitations, we were able to demonstrate that the COVID-19 pandemic led to a strong decline in the detection rate of newly diagnosed gynecological and breast cancers, which is in accordance with the findings of other subspecialities.

## Data Availability

Data available on request due to privacy/ethical restrictions.
